# Manufacturing and supply of face shields in hospital operation in case of unclear and confirmed COVID-19 infection status of patients

**DOI:** 10.1007/s00068-020-01392-3

**Published:** 2020-05-27

**Authors:** Jonas Neijhoft, Tobias Viertmann, Simon Meier, Nicolas Söhling, Sabine Wicker, Dirk Henrich, Ingo Marzi

**Affiliations:** 1 Department of Trauma, Hand and Reconstructive Surgery, Goethe University Frankfurt, Theodor-Stern-Kai 7, 60590 Frankfurt am Main, Germany; 2 Occupational Health Service, Goethe University, Frankfurt, Frankfurt am Main, Germany

**Keywords:** COVID-19, SARS-CoV-2, Corona, Severe acute respiratory syndrome coronavirus 2, 3D-printing, Health care workers protection, Traumatology

## Introduction

Initially appeared in the Chinese city of Wuhan, the novel respiratory disease COVID-19, which is caused by the, until this point, unknown coronavirus SARS-CoV-2, evolved to a pandemic with more than 850,000 infections in only 3 months [1].

One reason for this rapid spread arises from the high infectiousness of the virus, even in the incubation period of 2–14 days, whereby asymptomatic patients can pass it unwittingly. Additionally, the transmission happens easily through droplets or airborne infection, e.g. by contact with the conjunctiva [2, 3].

In a clinical context, there are situations where the treating physician has no knowledge about whether the patient is infected or not, for example in the emergency room. Health care workers therefore must protect themselves with personal protective equipment. As a face mask leaves the eyes and the facial skin unprotected, a face shield is required. Furthermore, it prevents health care workers from uncontrolled self-contamination by touching the face. Due to great demand and supply difficulties on the part of manufacturers, healthcare providers are facing the problem of hindered provisioning of protective equipment. Hence, the University Medical Center Frankfurt (Germany) developed an approach of using open source 3D printing technology [4, 5] to produce face shields in great quantities independently.

## Development

The requirements for a face shield used in a clinical context are shown in Table 1.

**Table 1:** Requirements a face shield must met

Effective protection of the face against droplets
Easy to disinfect
Comfortable to wear for a longer period of time
Fast in production
Durable
Low in price

**Fig. 1:**
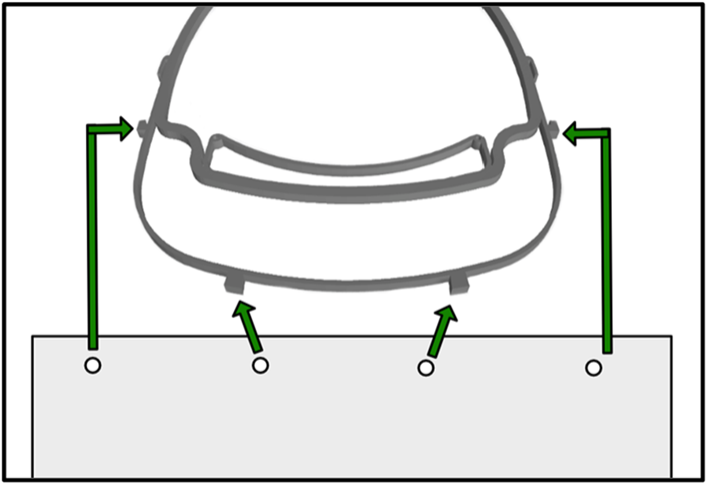
For assembly, a plastic sheet is punched with the extended ISO 838 4-hole format (also known as “888” or “3 × 8”) and the corresponding holes are clipped into the anchors of the frame [11]. After usage, the plastic sheet can be thrown away while the frame can be disinfected

**Fig. 2:**
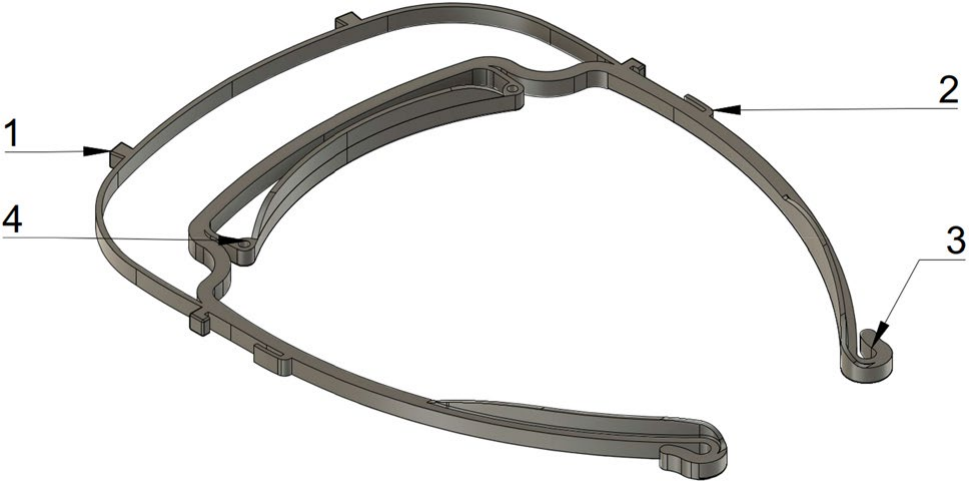
The distance of the visor to the head has been increased to make it comfortable to wear FFP-Masks. The floor in the area of the forehead rest was removed to increase wearing comfort. 1: Anchors for attaching the plastic sheet. 2: Brackets that hold the visor directly against the base part on the edges. 3: Tight loop at the end to hold the elastic band reliably. 4: Interfaces were added in order to install possible extensions (e.g. increased forehead rest)

A very simple concept to meet those requirements is a frame worn on the head out of a sanitizable material with a transparent plastic sheet attached to it as visor (Fig. 1). Optionally an elastic band can be connected to the hooks of the temple for a secure fit on the head (Fig. 2). Unfortunately, the 3D printable models of the frame that are currently available online did not meet all of those requirements, mainly in terms of comfort and time needed to print [6–8]. The face shield must be easy to handle and fast to put on and take off. Since polylactic acid (PLA) is both cheap, durable, sanitizable and easily processable, 3D printing of PLA is an eligible way to produce the frame [9, 10].

**Fig. 3:**
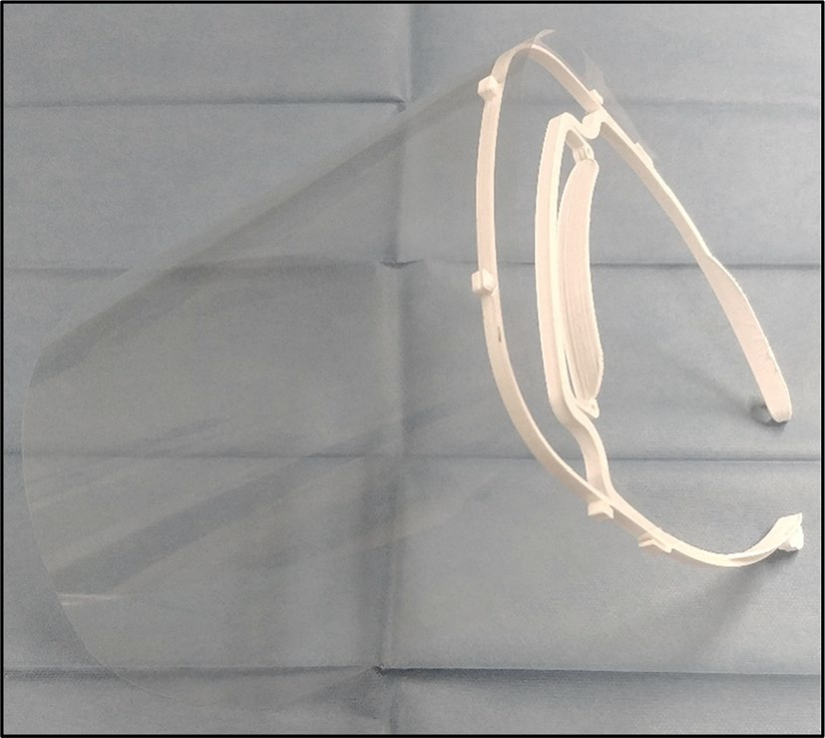
The assembled face shield

**Fig. 4:**
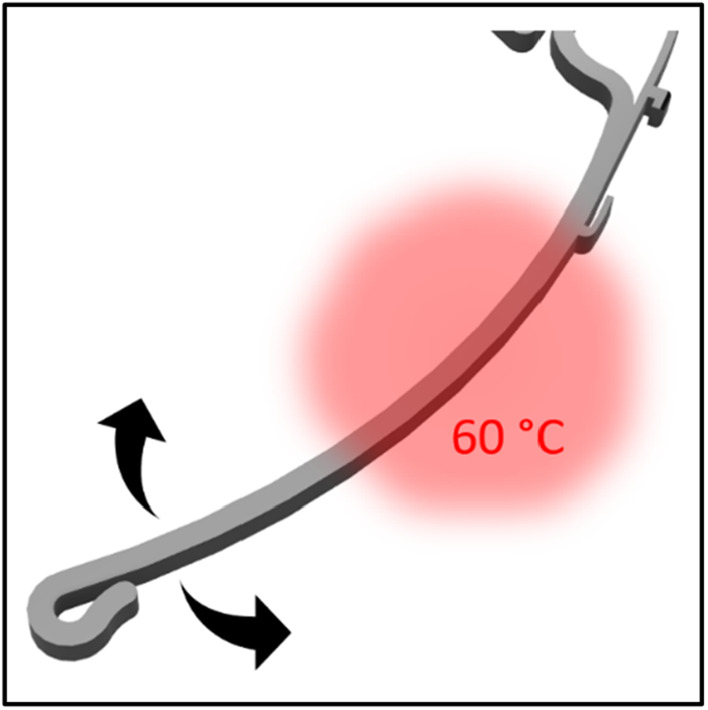
Heating the shown area under hot water makes further adjustment of the frame possible

The basic idea derives from a model found on the internet [8]. The following points were improved:

To increase the flexibility and in the same step shorten the printing time and price, the amount of filament that was used was decreased by cutting the height of the frame in half. Modifications to the anchor and forehead rest contribute to improved comfort even when worn for long periods of time (Figs. 2 and 3). Although the designs are produced in one size that should fit all head sizes, they can be easily adapted by pouring warm water on a specific point of the temple and bended to the perfect fit. (Fig. 4).

In highly infectious situations, the use of conventional FFP masks is needed; however, these masks protrude a few centimeters to the front. To prevent them from hitting the shield, the distance to the shield has been increased. As a result of this modification, it is also fogging up less quickly. However, one has to be aware, that the face shield does not replace a hood, glasses or face mask deliberately, it only serves as additional protection in case of patient contact. Depending on the area of application, adapters can be plugged in, for example, a shield closed at the top or an extended forehead rest (Fig. 2).

Production is ensured centrally in the hospital and decentral by production capacities by individuals, companies and other institutions.

**Fig. 5:**
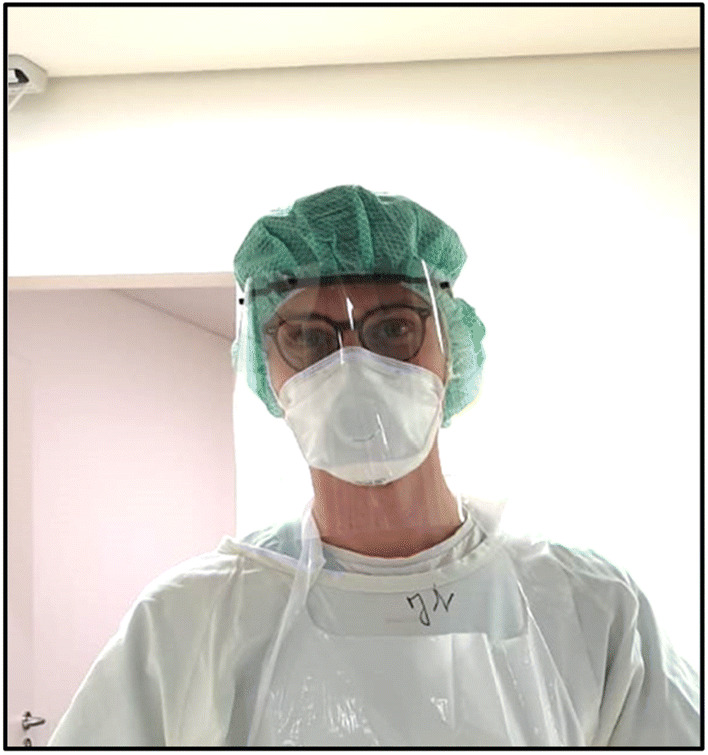
Face shield used in the emergency room

For quality assurance purposes, each printer prints in a different filament color. Thus, problems and signs of wear can be assigned to the respective printer afterwards. At the moment the staff of the University Medical Center of Frankfurt and in the future surrounding hospitals and emergency cars will also be equipped with the face shields (Fig. 5).

The shield is designed in a way that they can be produced with commercially available 3D printers. The 3D files as well as instructions are available in supplementary material.

Hopefully it will be possible to contain the spread of COVID-19 shielded together.

## Electronic supplementary material

Below is the link to the electronic supplementary material.

Supplementary material 1 (STL 804 kb)
